# Evaluation of Liofilchem Derma-SR-Screen 4-Well Agar Panels in Screening of Terbinafine and Itraconazole Susceptibility in Clinical *Trichophyton* Isolates

**DOI:** 10.3390/jof12040246

**Published:** 2026-03-27

**Authors:** Karin Meinike Jørgensen, Nissrine Abou-Chakra, Karen Marie Thyssen Astvad, Maiken Cavling Arendrup

**Affiliations:** 1 Unit of Mycology, Statens Serum Institute, 2300 Copenhagen, Denmark; kmj@ssi.dk (K.M.J.); nac@ssi.dk (N.A.-C.); kaas@ssi.dk (K.M.T.A.); 2 Department of Clinical Microbiology, Rigshospitalet, 2100 Copenhagen, Denmark; 3 Department of Clinical Medicine, University of Copenhagen, 2100 Copenhagen, Denmark

**Keywords:** *Trichophyton*, terbinafine, itraconazole, antifungal resistance, susceptibility screening, screening agar

## Abstract

The aim of this paper is to evaluate the performance of the Derma-SR-screen agar for accurate discrimination between susceptible and non-susceptible clinical *Trichophyton* isolates. Consecutive *Trichophyton* isolates, received for identification and susceptibility testing, were screened for terbinafine and itraconazole resistance using Liofilchem Derma-SR-screen 4-well panels alongside EUCAST reference testing (E.Def 11.0). EUCAST tentative ECOFFs (terbinafine: *T. rubrum* 0.03 mg/L; *T. indotineae* 0.125 mg/L; itraconazole: both species: 0.25 mg/L) were applied for wild-type/non-wild-type classification. Plates were evaluated after 5 days of incubation at 25 °C, with growth graded 0-+++. Faint growth (+) was disregarded. All isolates underwent *sqle* sequencing. Forty isolates were included; 25 were non-wild-type harbouring Sqle alterations (F397I (number (n) = 1), F397L (n = 17), L393F (n = 3), L393S (n = 1), and Q408L (n = 3)). On day 5, 21 isolates reached +++ growth in the control well; a further 10 reached this level on day 6. The remaining isolates reached a ++/+++ score after 5/6 days (n = 7/n = 2). The 0.125 mg/L terbinafine agar correctly identified 7/8 non-wild-type *T. indotineae* isolates (4/5 F397L and 3/3 Q408L alterations), all 17 non-wild-type and eight wild-type *T. rubrum* isolates, as well as the five wild-type isolates of other *Trichophyton* spp. The 0.016 mg/L agar correctly identified all 17 non-wild-type *T. rubrum* isolates, but misclassified 2/8 wild-type isolates as non-wild-type. All isolates were wild-type to itraconazole and correctly identified. The Derma-SR-screen agar resulted in correct classification of 24/25 (96%) *sqle* mutant *T. indotineae* and *T. rubrum* isolates. Two wild-type *T. rubrum* isolates grew at the 0.016 mg/L terbinafine agar suggesting possible reduced agar potency at this concentration.

## 1. Introduction

Treatment of refractory dermatophytosis, particularly due to terbinafine-resistant *Trichophyton* species, is a worldwide concern. One major route of resistance acquisition is linked to the recent and rapid global spread of the newly recognised species *T. indotineae,* which is characterised by a high rate of terbinafine resistance. It was first reported in India under the name *T. interdigitale* [1,2], subsequently described as *T. mentagrophytes* VIII [3], and later recognised as the cause of treatment-refractory dermatophytosis in other regions, often with epidemiologic links to the Indian subcontinent [4]. Supporting this, a recent study analysing isolates collected over a decade (2014–2023) from the UK, France, Ireland, Canada and India found no clear geographical clustering of isolates, confirming the rapid transcontinental spread of *T. indotineae* from its probable centre of diversity in Asia [5]. The second most common cause of terbinafine-resistant dermatophytosis is *T. rubrum*. In Denmark, this species currently represents the leading cause of treatment-refractory dermatophytosis [6]. However, recent observations suggest that *T. indotineae* may soon overtake this position [7].

Susceptibility testing has been standardised by CLSI and EUCAST [8,9]; however, testing remains time-consuming and technically challenging. Microdilution plates are not commercially available for dermatophyte testing, and the slow growth of dermatophytes often complicates or delays preparation of sufficient inoculum. In addition, contamination by more rapidly growing organisms may occur, particularly with the CLSI method, which relies on a medium that is not selective for dermatophytes [8,9].

To facilitate detection of terbinafine and itraconazole resistance in *Trichophyton* species, EUCAST has developed an agar-based screening method validated in a multicentre study using a blinded strain collection comprising wild-type and *sqle* mutant non-wild-type isolates [10].

Recently, the commercial kit Derma-SR-screen (RUO) (Liophilchem Nordics ApS, Copenhagen, Denmark) has been introduced, potentially facilitating susceptibility screening in clinical laboratories that perform dermatophyte cultures. Here, we evaluated the performance of these plates using consecutive clinical samples with EUCAST MIC testing and *sqle* sequencing as the reference standard.

## 2. Materials and Methods

Forty consecutive and unrelated consecutive clinical *Trichophyton* isolates from samples referred to our reference laboratory for identification and susceptibility testing during the second half of 2025 were included. Culturing was performed using Yeast Glucose Chloramphenicol Medium (YGC) Agar plates (Oxoid, Thermo Fischer, Waltham, MA, USA) supplemented in-house with 500 microliters of a suspension of sterile water and Cycloheximide Ready-Made Solution 100 mg/mL in DMSO (Merck life Science, Søborg, Denmark (hereafter Merck, Rahway, NJ, USA)) for a final agar cycloheximide concentration of 300 mg/L. Plates were incubated at 25 °C for up to 4 weeks. Identification, ITS sequencing and *SQLE* sequencing were performed as previously described [6].

For susceptibility screening and EUCAST testing, colonies were covered with sterile water supplemented with 0.1% Tween-20, carefully rubbed, and the suspension aspirated in a sterile syringe and filtered through an 11 µm filter (Nylon Net Filter, 11 µmNY11, Millipore, Carrigtwohill, Ireland, placed in a Swinnex Filter Holder, 25 mm, MM_NF-SX0002500, www.MerckMillipore.com) and adjusted by an OD metre (Densimat, BioMerieux Denmark, Lautruphøj 1, 2750 Ballerup) to the target concentration 2 × 10^6^ to 5 × 10^6^ CFU/mL as described in the EUCAST E.Def 11.0 reference method [9]. Following inoculation of Derma-SR-screen 4-well panels (Liophilchem Nordics ApS, Copenhagen, Denmark) with 25 µL per well, the inoculum suspensions were diluted 1:10 with sterile distilled water to obtain the final working inoculum of 2 × 10^5^ to 5 × 10^5^ CFU/mL appropriate for MIC determination according to EUCAST E.Def 11.0, to allow direct comparison of the two methods using the same inoculum suspension. Utensils for the MIC determination included Microtiter plates (Thermo Scientific™ Nunc™ MicroWell™ 96-Well, Nunclon Delta-Treated, Flat-Bottom Microplate, catalogue no. 161093; Fisher Scientific Biotech Line ApS, Roskilde, Denmark), double-concentrated RPMI 1640 buffered with 3-(*N*-morpholino) propanesulfonic acid (MOPS) and supplemented with 2% glucose (SSI Diagnostica, Hillerød, Denmark), DMSO (Merck), and terbinafine and itraconazole (Merck; 0.004 to 4 mg/L). Cycloheximide (Merck, ready-made solution 100 mg/mL in DMSO) and chloramphenicol (Merck) were added to the inoculum solution as per protocol (final concentrations in the inoculated susceptibility plate, 50 mg/L and 300 mg/L, respectively) [9]. Plates were read using a 50% inhibition endpoint compared to antifungal free control wells, using a spectrophotometer (Biotek EPOCH2 (Halby, Brøndby, Denmark) 490 nm wavelength). Incubation time at 25 °C was 5(–7) days (preferentially 5 days) [9]. Tentative ECOFFs (TECOFFs) were applied for wild-type/non-wild-type classification: Terbinafine; *T. indotineae* TECOFF = 0.125 mg/L and *T. rubrum* TECOFF = 0.03 mg/L; and itraconazole both species TECOFF = 0.25 mg/L [9].

The 4-well panels were incubated alongside the microtitre plates for 5 days at 25 °C before evaluation. If growth was insufficient in the growth control wells of the 4-well panel or in the microtitre plate, both plate types were further incubated. Growth was scored as follows: 0, no growth/nothing visible on the agar; (+), trace material on the agar, consistent with either residual inoculum or early growth; +, very little but evident growth; ++, evident to significant growth but no visible hyphae; and +++, significant growth with visible hyphae.

Fisher’s exact test was used to compare the degree of growth across terbinafine wild-type and non-wild-type isolates (Graph Pad Prism 10.0.2).

Incorrect susceptibility classification results were defined as follows: very major error (VME)—non-wild-type isolates misclassified as wild-type by the Derma-SR-screen panel; major error (ME)—wild-type isolate misclassified as non-wild-type by the Derma-SR-screen panel.

## 3. Results

The forty *Trichophyton* isolates included nine *T. indotineae*, one *T. interdigitale*, three *T. mentagrophytes*, 26 *T. rubrum*, and one *T. tonsurans*. Fifteen had wild-type terbinafine susceptibility and *sqle*. Twenty-five were non-wild-type and harboured the following Sqle alterations: L393F (n = 3), L393S (n = 1), F397I (n = 1), F397L (n = 17) and Q408L (n = 3) (Table 1). For itraconazole, the MIC ranges were as follows: *T. indotineae* 0.008–0.125 mg/L, *T. interdigitale* 0.06 mg/L, *T. mentagrophytes* 0.03–0.125 mg/L, *T. rubrum* 0.008–0.25 mg/L and *T. tonsurans* 0.016 mg/L. All *T. indotineae* and *T. rubrum* were wild-type to itraconazole. Finally, itraconazole MICs against the few *T. interdigitale*, *T. mentagrophytes* and *T. tonsurans* were all ≤ 0.06 mg/L, suggesting low likelihood of acquired resistance, although formal EUCAST TECOFFs/BPs have not been set for these species.

All isolates grew on the antifungal free control agars of the Derma-SR-screen panels, although species-specific differences were noticed. The strongest growth was seen for *T. indotineae* (9/9 scored +++), followed by *T. rubrum* (20/26 scored +++), with no difference between wild-type and mutants (7/9 versus 13/17, Fisher’s exact test *p* > 0.999). For the remaining species, *T. interdigitale*, *T. mentagrophytes* and *T. tonsurans,* strong growth was observed for 2/5 isolates.

Following the EUCAST recommendation to rely on the high (0.125 mg/L) terbinafine concentration well for the interpretation of *T. indotineae* susceptibility, 7/8 resistant mutants were correctly classified as non-susceptible, whereas one isolate (MIC > 4) harbouring the F397L alteration presented (+) growth comparable to the wild-type *T. indotineae* isolate (MIC 0.03 mg/L) and constituted a VME. For *T. rubrum*, EUCAST recommends the low (0.016 mg/L) terbinafine concentration well to be used for classification. Here, 17/17 resistant mutants were correctly classified as non-susceptible. However, two out of eight wild-type isolates (both MIC 0.016 mg/L) were also misclassified as non-susceptible due to (+)/+ growth on the terbinafine 0.016 mg/L agar constituting MEs. If, on the other hand, only the high terbinafine well was considered for all species, 7/8 *T. indotineae* and 17/17 *T. rubrum* non-wild-type isolates were correctly classified as such (one very major error among the 25 *sqle* mutant isolates (4%)).

The *T. interdigitale*, *T. mentagrophytes* and *T. tonsurans* isolates were all categorised as terbinafine wild-type (0-(+) growth) on both terbinafine concentration agars, although agar screening is not validated by EUCAST for these species.

With respect to itraconazole susceptibility, MICs were as follows: MIC_50_ 0.06 mg/L, modal MIC 0.125 mg/L and range 0.008–0.25 mg/L with no apparent species-specific differences (Table 2). Trace growth (+) was observed for 18/40 isolates, including 4/9 *T. indotineae*, 1/1 *T. interdigitale*, 12/26 *T. rubrum* and 1/1 *T. tonsurans*, again with no apparent species-specific difference.

Contamination was seen for one wild-type *T. rubrum* isolate in the terbinafine 0.016 mg/L agar well and for two wild-type *T. rubrum* isolates in the terbinafine 0.125 mg/L agar wells, but not in the microtitre MIC plates.

## 4. Discussion

Following the concerning rise in terbinafine refractory dermatophytosis reported world-wide, patient-near susceptibility testing has become urgently needed. The development of a standardised microdilution MIC method for dermatophytes and associated TECOFFs has been a major step forward, although MIC testing is not straightforward or easily implementable in routine laboratories [8,9]. With the growing research and characterisation of resistance mechanisms in dermatophytes, molecular resistance detection has become available by commercial methods. However, tests such as the DermaGenius (PathoNostics, NL) include only the most common resistance mutations involving the two loci L393 and F397, which in our setting would miss 12% (3/25) of the resistant isolates (carrying Q408L). Therefore, such tests can allow early detection of resistance but cannot confirm susceptibility. On the contrary, EUCAST agar screening tests for detection of azole resistance in *A. fumigatus*, echinocandin resistance in *Aspergillus* and most recently terbinafine and itraconazole resistance in *Trichophyton* have the potential to fill this need [10,11]. The initial evaluation of the first commercially available Derma-SR-screen kit (RUO) is an important step in bringing susceptibility screening closer to the patient.

We found a VME rate of 4% for terbinafine screening of *T. indotineae* and *T. rubrum* isolates (using the high and low terbinafine well, respectively) with the Derma-SR-screen plates. However, 2/8 wild-type *T. rubrum* showed weak growth (+)/+ at the terbinafine 0.016 mg/L well and were thus misclassified as resistant (ME). Therefore, the testing of more wild-type *T. rubrum* isolates is needed to clarify the proportion of MEs, and to understand if isolates with (+)/+ need repeat or additional testing before being reported to avoid overestimating resistance. This is of particular importance if the screening agar is implemented more widely in settings with a lower prevalence of resistance than in our reference laboratory. Moreover, it is important that laboratories include testing of the EUCAST quality control strains (*T. rubrum* SSI-7583 and *T. indotineae* SSI-9363) available from the CCUG Culture collection University of Gothenburg (www.ccug.se) in parallel to assist correct differentiation of wild-type and non-wild-type organisms and correct performance of the agars.

The overall growth patterns of non-susceptible isolates in the 0.016 mg/L and 0.125 mg/L terbinafine wells were notably similar despite an 8-fold difference in terbinafine concentration. We believe this observation warrants further investigation and confirmation of the exact concentration and stability of terbinafine in the agars.

Our study has limitations. First, the number of clinical isolates included was limited, particularly of wild-type isolates of *T. indotineae* and of other species such as *T. interdigitale*, *T. mentagrophytes* and *T. tonsurans*. Second, we did not find any isolates with a non-wild-type phenotype for itraconazole, and therefore we cannot evaluate the performance of the Derma-SR-screen 4-well plates for detection of itraconazole resistance. On the other hand, it was somewhat reassuring that 18/40 isolates displayed trace growth at the itraconazole well, as this may suggest that the concentration chosen may be appropriate for allowing isolates with elevated MICs to grow properly. Strengths include the head-to-head comparison of reference EUCAST testing and agar screening based upon the same culture and inoculum preparation, and that all non-susceptible isolates were confirmed to carry recognised resistance-conferring target gene mutations and that the most prevalent mutations were represented.

In conclusion, this pilot study on the performance of the new Derma-SR-screen panel for detection of terbinafine-resistant *Trichophyton* species suggests that it is a promising tool for *T. rubrum* and for *T. indotineae,* potentially feasible for implementation in clinical laboratories that culture dermatophytes. Yet additional studies verifying drug stability and including more wild-type organisms and, if possible, additional resistance mechanisms are warranted.

## Figures and Tables

**Table 1:** Growth scores for the tested isolates sorted by species and SQLE type. Terbinafine (TRB) MIC ranges are included for comparison. No or (+) growth on drug-containing agars were not taken into considerations (greyed out in the table). The agar concentrations recommended for terbinafine screening of *T. indotineae* and *T. rubrum* are indicated by the boxes.

Species SQLE Type (*n*.)	Derma-SR-Screen Agar Well *																TRB MIC ** Range (mg/L)
	Growth Control			TRB 0.016 mg/L							TRB 0.125 mg/L						
	++/+++	+++		(+)	(+)/+	+	+/++	++	++/+++		0	(+)	+	+/++	++		
*T. indotineae*																	
F397L (5)		5			1		1	3				**1**		2	2		4–>4
Q408L (3)		3					1	2						3			0.5–1
Wild-type (1)		1				1						1					0.03
																	
*T. interdigitale*																	
Wild-type (1)	1			1								1					0.016
																	
*T. mentagrophytes*																	
Wild-type (3)	2	1		1		1		1			3						0.016–0.06
																	
*T. rubrum*																	
L393F (3)	1	2					2	1					1	1	1		>4
L393S (1)		1						1							1		1
F397I (1)		1						1							1		1
F397L (12)	3	9				1		10	1						12		1–>4
Wild-type (9) ***	**2**	7		6	2						3	4					0.008–0.03
																	
*T. tonsurans*																	
Wild-type (1)		1		1								1					0.016
In total (40)	9	31		9	3	3	4	19	1		6	8	1	6	17		

* Growth was scored as follows: 0, no growth/nothing visible on the agar; (+), trace material on the agar, consistent with either residual inoculum or early growth; +, very little but evident growth; ++, evident to significant growth but no visible hyphae; and +++, significant growth with visible hyphae. ** Terbinafine MICs were obtained by EUCAST E.Def 11.0 microdilution method. *** Contamination was seen for one isolate in TRB 0.016 mg/L and for two isolates in TRB 0.125 mg/L, which were excluded from analysis. Bold and underlined font indicates very major errors (resistant isolates classified as susceptible), underlined font as major errors (wild-type isolates classified as resistant).

**Table 2:** Growth scores for the tested isolates sorted by species and itraconazole (ITC) MIC (mg/L).

Species ITC MIC			Growth Control				ITC 1 mg/L Well			
	*N*		++/+++	+++			0	0/(+)	(+)	
*T. indotineae*	9									
0.008				2			1		1	
0.016				2			2			
0.03				3					3	
0.06										
0.125				2			2			
										
*T. interdigitale*	1									
0.06			1						1	
										
*T. mentagrophytes*	3									
0.03			1				1			
0.06										
0.125			1				2			
										
*T. rubrum*	26									
0.008				1			1			
0.016				2			2			
0.03				3			3			
0.06			2	5			1	1	5	
0.125			3	6			6		3	
0.25			1	3			1		3	
										
*T. tonsurans*	1									
0.016				1					1	
In total	40		9	31			22	1	17	

## Data Availability

The original contributions presented in this study are included in the article. Further inquiries can be directed to the corresponding author.

## References

[1] Rudramurthy S.M., Shankarnarayan S.A., Dogra S., Shaw D., Mushtaq K., Paul R.A., Narang T., Chakrabarti A. (2018). Mutation in the Squalene epoxidase gene of Trichophyton interdigitale and Trichophyton rubrum associated with allylamine resistance. Antimicrob. Agents Chemother..

[2] Singh A., Masih A., Khurana A., Singh P.K., Gupta M., Hagen F., Meis J.F., Chowdhary A. (2018). High terbinafine resistance in Trichophyton interdigitale isolates in Delhi, India harbouring mutations in the squalene epoxidase gene. Mycoses.

[3] Nenoff P., Verma S.B., Vasani R., Burmester A., Hipler U., Wittig F., Krüger C., Nenoff K., Wiegand C., Saraswat A. (2019). The current Indian epidemic of superficial dermatophytosis due to Trichophyton mentagrophytes—A molecular study. Mycoses.

[4] Nenoff P., Verma S.B., Ebert A., Süß A., Fischer E., Auerswald E., Dessoi S., Hofmann W., Schmidt S., Neubert K. (2020). Spread of terbinafine-resistant trichophyton mentagrophytes type VIII (India) in Germany–“the tip of the iceberg?”. J. Fungi.

[5] Rhodes J., Hui S.T., Dellière S., Summerbell R.C., A Scott J., Kaur A., Barton R.C., Leitao R., Hemmings S., Goiriz R. (2026). Emerging terbinafine-resistant Trichophyton indotineae between 2018 and 2023: A multinational genomic epidemiology study. Lancet Microbe.

[6] Astvad K.M.T., Hare R.K., Jørgensen K.M., Saunte D.M.L., Thomsen P.K., Arendrup M.C. (2022). Increasing Terbinafine Resistance in Danish Trichophyton Isolates 2019–2020. J. Fungi.

[7] Astvad K.M.T., Jørgensen K.M., Abou-Chakra N., Meletiadis J., Saunte D.M.L., Antsupova V.S., Lützen L., Kristensen L., Gertsen J.B., Arendrup M.C. Antifungal Resistance in Trichophyton Isolates from Denmark a 2021–2024 Update. Proceedings of the Trends in Medical Mycology, TIMM.

[8] CLSI (2017). Reference Method for Broth Dilution Antifungal Susceptibility Testing of Filamentous Fungi.

[9] Arendrup M.C., Kahlmeter G., Guinea J., Meletiadis J. (2021). Subcommittee on Antifungal Susceptibility Testing (AFST) of the ESCMID European Committee for Antimicrobial Susceptibility Testing (EUCAST). How to: Perform antifungal susceptibility testing of microconidia-forming dermatophytes following the new reference EUCAST method E.Def 11.0, exemplified by Trichophyton. Clin. Microbiol. Infect..

[10] Meletiadis J., Siopi M., Jørgensen K.M., Escribano P., van de Lee H., Buil J.B., Friberg N., Guinea J., Arendrup M.C. (2025). Multicentre evaluation of a EUCAST-based agar-screening method for terbinafine and itraconazole susceptibility of *Trichophyton* spp.. Clin. Microbiol. Infect..

[11] Meletiadis J., Guinea J., Arikan-Akdagli S., Friberg N., Kahlmeter G., Arendrup M.C. EUCAST Definitive Document E.Def 10.3. Screening Method for the Detection of Azole and Echinocandin Resistance in Aspergillus Using Antifungal-Containing Agar Plates, Exemplified by *A. fumigatus* 2025. https://www.eucast.org/fungi-afst/methodology-and-instructions/ast-of-moulds/.

